# Successful Laparoscopic Resection of Biliary Cystadenoma: A Case Report

**DOI:** 10.7759/cureus.51784

**Published:** 2024-01-07

**Authors:** Hadeel Alosaimi, Rana Alotaibi, Razan Almuwallad, Maha Aljohani, Ahmad Abdulfatah

**Affiliations:** 1 General Practice, University of Tabuk, Tabuk, SAU; 2 General Surgery, University of Tabuk, Tabuk, SAU; 3 Surgery, Dallah Hospital, Riyadh, SAU

**Keywords:** abdominal pain, laparoscopy, computed tomography, hepatic lesion, biliary cystadenoma

## Abstract

Biliary cystadenomas are rare hepatic lesions originating from the biliary epithelium. We present the case of a 32-year-old female who presented with persistent right upper quadrant abdominal pain, prompting a thorough investigation. Mildly elevated liver enzymes were noted on laboratory testing. Imaging studies, including a contrast-enhanced CT scan, revealed a 14 cm multiloculated cystic lesion in the right lobe of the liver. A biliary cystadenoma was confirmed, leading to a collaborative decision for laparoscopic resection. Intraoperative findings and histopathological examination supported the diagnosis, and the patient had an uneventful recovery postoperatively. This case report underscores the clinical complexity of biliary cystadenomas and highlights the successful multidisciplinary management of a young patient through laparoscopic resection. The case contributes valuable insights into the diagnostic and therapeutic challenges associated with these uncommon hepatic lesions.

## Introduction

Biliary cystadenomas are rare hepatic neoplasms that predominantly arise from the biliary epithelium. These tumors, while infrequently encountered, pose unique challenges in diagnosis and management. Initially described by Edmondson in 1958, biliary cystadenomas account for a small fraction of cystic liver lesions, with an incidence of less than 5% among all primary liver cysts [1]. Typically observed in middle-aged females, these lesions often present with nonspecific symptoms, making their diagnosis challenging. The etiology of biliary cystadenomas remains unclear, and their pathogenesis is thought to involve complex interactions between genetic factors and hormonal influences [2]. Given the rarity of these tumors, standardizing diagnostic and management approaches remains a subject of ongoing refinement. This case report discusses the clinical presentation, diagnostic evaluation, and successful management of a young female patient with a biliary cystadenoma in the liver. Through an integrative and multidisciplinary approach, we aim to contribute to the evolving understanding of these uncommon hepatic lesions and emphasize the importance of accurate diagnosis and timely intervention. 

## Case presentation

A 32-year-old female patient presented to our medical facility with a chief complaint of persistent right upper quadrant abdominal pain for the past six months. The pain was dull in nature, intermittent, and associated with occasional nausea. There was no history of fever, jaundice, or weight loss. Her past medical history was unremarkable, with no significant surgical interventions or known chronic illnesses. The patient reported no history of alcohol consumption or tobacco use. Family history revealed no significant liver or gastrointestinal disorders.

On physical examination, the patient appeared generally well with no signs of distress. Vital signs were within normal limits. Abdominal examination revealed tenderness in the right upper quadrant without rebound or guarding. Murphy's sign was negative. No hepatomegaly or splenomegaly was appreciated. The remainder of the systemic examination was unremarkable.

Laboratory investigations were conducted to evaluate liver function, complete blood count, and tumor markers. The results demonstrated a mild elevation in liver enzymes, with alanine transaminase at 78 U/L (normal range: 7-56 U/L) and aspartate transaminase at 58 U/L (normal range: 13-39 U/L). Alkaline phosphatase was within normal limits at 90 U/L (normal range: 44-147 U/L), as were total bilirubin, direct bilirubin, and indirect bilirubin. Tumor markers, including alpha-fetoprotein and carcinoembryonic antigen, were within normal ranges (Table 1).

**Table 1:** Laboratory test results with reference ranges The indicator key denotes the patient's laboratory results with the reference ranges: L for Low, N for Normal, and H for High.

Lab Test	Result	Reference Range	Indicator
Alanine Transaminase	78 U/L	7-56 U/L	H
Aspartate Transaminase	58 U/L	13-39 U/L	H
Alkaline Phosphatase	90 U/L	44-147 U/L	N
Total Bilirubin	0.8 mg/dL	0.2-1.2 mg/dL	N
Direct Bilirubin	0.3 mg/dL	0-0.3 mg/dL	N
Indirect Bilirubin	0.5 mg/dL	0.1-1.0 mg/dL	N
Albumin	4.0 g/dL	3.5-5.5 g/dL	N
Total Protein	7.2 g/dL	6.0-8.3 g/dL	N
Blood Urea Nitrogen	15 mg/dL	7-20 mg/dL	N
Creatinine	0.8 mg/dL	0.6-1.1 mg/dL	N
Glucose	85 mg/dL	70-99 mg/dL	N
Lipase	50 U/L	13-60 U/L	N
Amylase	40 U/L	30-110 U/L	N
White Blood Cell Count	8.0 K/µL	4.0-11.0 K/µL	N
Hemoglobin	13.5 g/dL	12.0-15.5 g/dL	N
Platelet Count	320 K/µL	150-450 K/µL	N
Red Blood Cell Count	5.0 M/µL	4.5-5.5 M/µL	N
Mean Corpuscular Volume	90 fL	80-100 fL	N
Alpha-fetoprotein	8 ng/mL	< 10 ng/mL	N
Carcinoembryonic Antigen	3.0 ng/mL	< 5.0 ng/mL	N

**Figure 1:**
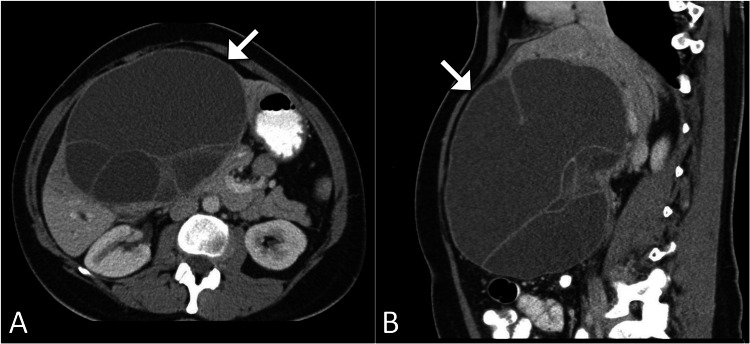
Axial (A) and sagittal (B) CT images depicting a prominent multiloculated hepatic lesion (indicated by arrow) in the right lobe

Further characterization was pursued with a contrast-enhanced CT scan of the abdomen. The scan confirmed the presence of a multiloculated cystic lesion in the right lobe of the liver, measuring approximately 14 cm in diameter, with internal septations and enhancing mural nodules. The imaging findings were consistent with a biliary cystadenoma. Given the radiological characteristics and the patient's clinical presentation, the most likely diagnosis was a biliary cystadenoma (Figure 1). A liver biopsy was not pursued due to the risk of cyst rupture and potential complications.

**Figure 2:**
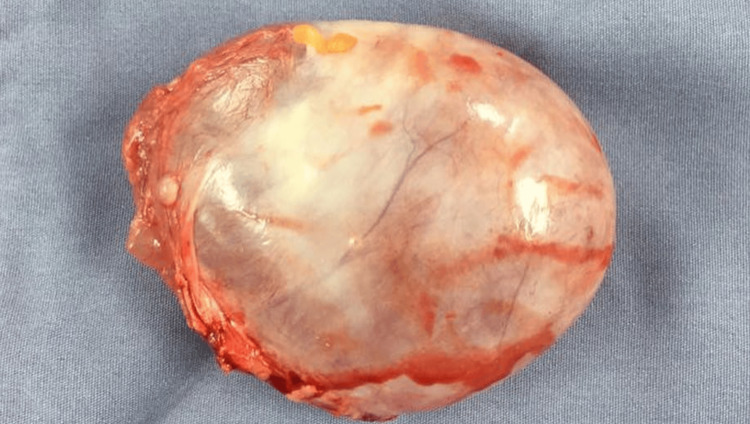
Postoperative image illustrating the successfully resected hepatic lesion

**Figure 3:**
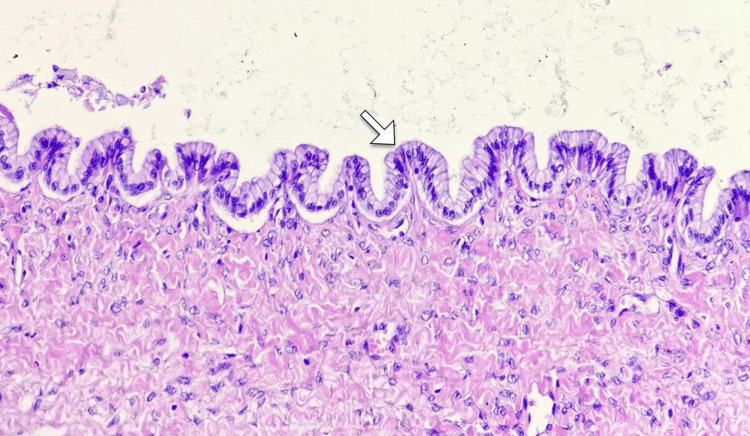
Histopathological examination revealing cuboidal epithelium (arrow), providing insight into the characteristic features of the biliary cystadenoma

The patient was managed conservatively with pain control, and surgical consultation was sought for definitive management. A multidisciplinary approach involving hepatobiliary surgeons, radiologists, and anesthesiologists was undertaken. The patient underwent a successful laparoscopic resection of the biliary cystadenoma. Intraoperative findings included a large cystic mass adherent to the liver parenchyma (Figure 2). The pathology report confirmed the diagnosis of a biliary cystadenoma with no evidence of malignancy (Figure 3).

Postoperatively, the patient had an uneventful recovery and was discharged on postoperative day four. Regular follow-up appointments were scheduled to monitor for any signs of recurrence or complications. Serial laboratory investigations were planned to ensure the patient's long-term well-being and to detect any potential recurrence promptly.

## Discussion

Biliary cystadenomas represent a distinctive subset of hepatic cystic lesions with unique clinical and pathological characteristics. This case report contributes to the existing body of literature by detailing the clinical presentation, diagnostic challenges, and successful management of a biliary cystadenoma in a young female patient. The rarity of this tumor emphasizes the significance of individual case reports in enhancing our understanding of the disease spectrum and refining diagnostic and therapeutic strategies.

The clinical presentation of biliary cystadenomas often mimics that of other hepatobiliary conditions, making their diagnosis challenging [2-4]. In this case, the patient presented with persistent right upper quadrant pain and a mild elevation in liver enzymes. These nonspecific symptoms underscore the importance of considering biliary cystadenomas in the differential diagnosis of hepatic lesions, particularly in young individuals without a significant medical history.

Accurate preoperative diagnosis remains elusive due to the lack of specific clinical and radiological features. While imaging studies, such as contrast-enhanced CT scans, provide valuable insights, the definitive diagnosis often requires histopathological examination [3-5]. In our case, a multidisciplinary approach, involving hepatobiliary surgeons and radiologists, was pivotal in guiding the decision-making process and optimizing patient outcomes.

The management of biliary cystadenomas poses unique challenges. Conservative approaches, such as cyst aspiration and sclerotherapy, have been explored, but surgical resection remains the mainstay of treatment [2,5]. In our case, laparoscopic resection was successfully employed, highlighting the feasibility and safety of minimally invasive techniques in managing these lesions. The histopathological examination confirmed the diagnosis of a biliary cystadenoma without evidence of malignancy. The absence of malignancy in this case aligns with the generally indolent nature of biliary cystadenomas; however, the potential for malignant transformation necessitates vigilant postoperative surveillance [3,5].

## Conclusions

In conclusion, this case report sheds light on the clinical intricacies, diagnostic challenges, and successful management of a biliary cystadenoma in a young female patient. The presented case emphasizes the importance of considering biliary cystadenomas in the differential diagnosis of hepatic lesions, especially when faced with nonspecific symptoms and a mild elevation in liver enzymes. The successful utilization of laparoscopic resection highlights the feasibility of minimally invasive approaches in managing these lesions. While the indolent nature of biliary cystadenomas was corroborated in this case, the potential for malignant transformation necessitates long-term surveillance.
